# Effects of Shenmai injection and its bioactive components following ischemia/reperfusion in cardiomyocytes

**DOI:** 10.3892/etm.2015.2662

**Published:** 2015-07-28

**Authors:** LI-FANG YE, YA-RU ZHENG, LI-HONG WANG

**Affiliations:** 1 Department of Cardiology, First Affiliated Hospital, Medical College of Zhejiang University, Hangzhou, Zhejiang 310003, P.R. China; 2 Department of Cardiology, People's Hospital of Zhejiang Province, Hangzhou, Zhejiang 310014, P.R. China

**Keywords:** Shenmai injection, ischemia/reperfusion, total saponins of *Panax ginseng*, phospholamban, ginsenoside Rg1, intracellular calcium

## Abstract

The aim of the present study was to determine whether the myocardial protective function of Shenmai injection (SM) during ischemia/reperfusion (I/R) is attributable to its regulation of intracellular calcium (Ca^2+^) and phospholamban (PLB) levels. Cultured neonatal Sprague Dawley rat cardiomyocytes were used to compare the effects of normoxia, total saponins of *Panax ginseng* (TSPG), ginsenoside Rg1 (Rg1) and SM treatments in rat myocardial cells following I/R. For each of these treatment groups, the mRNA and protein levels of PLB and the sarco/endoplasmic reticulum Ca^2+^ ATPase (SERCA) were evaluated, in addition to the cytoplasmic Ca^2+^ concentration [Ca^2+^]_i_ and the rate of apoptosis. The results indicated that I/R markedly decreased phosphorylated PLB and SERCA expression and that SM was able to mitigate this effect, while TPSG and Rg1 were not. Furthermore, SM appeared to prevent aberrant apoptosis and restore the depleted [Ca^2+^]_i_ resulting from I/R. The protective efficacy of SM against heart disease following I/R may, therefore, be due in part to its effect on intracellular Ca^2+^ homeostasis. SM may exert its protective effects by relieving PLB inhibition, and the pharmacodynamic actions of SM appear to be significantly more effective than those of its bioreactive components, TPSG and Rgl.

## Introduction

Human ischemic heart disease is the cause of 13.2% of all mortality worldwide (1). The upregulation of apoptosis and cardiac systolic dysfunction in diseased heart tissue are considered to contribute significantly to disease development and ultimate heart failure (2). Furthermore, a previous study suggested that the maintenance of intracellular calcium concentration [Ca^2+^]_i_ is critical for normal myocardial function (3). The major proteins responsible for maintaining intracellular Ca^2+^ homeostasis throughout excitation-contraction cycling in cardiomyocytes include sarco/endoplasmic reticulum Ca^2+^-ATPase (SERCA) and the SERCA regulatory protein phospholamban (PLB) (4). In its dephosphorylated state, PLB inhibits the uptake of Ca^2+^ by SERCA. By contrast, PLB phosphorylation by cAMP-dependent protein kinase A (PKA) at Ser16 and by Ca^2+^-calmodulin-dependent protein kinase II at Thr17 relieves this inhibitory inﬂuence on SERCA and thereby increases Ca^2+^ uptake by the sarcoplasmic reticulum (SR) (5). The phosphorylation of PLB, primarily by PKA at Ser16, is known to be associated with enhanced critical left ventricular functions, such as contractility and relaxation (6). The results of our previous studies showed that the induction of acute myocardial infarction in rats resulted in a significant downregulation of SERCA mRNA and protein expression and, conversely, caused a significant upregulation of PLB mRNA and protein expression (7,8). We therefore hypothesize that aberrant cardiac contractile function partially contributes to these observed effects, but it would be useful to determine the mechanism used by myocardial cells to stabilize the [Ca^2+^]_i_. In addition, it would be beneficial to investigate whether the regulation of PLB phosphorylation is sufficient to reduce cell apoptosis and improve cardiac systolic function. Performing these investigations would likely provide crucial insight into methods for improving the treatment of coronary heart disease.

Shenmai injection (SM), derived from the shendong drink described in the Zheng Yin Mai Zhi (which translates as ‘Pattern, Cause, Pulse and Treatment’) by Qin Changyu of the Ming dynasty (9), has been widely applied in recent years to treat qi-yin (10–14). The term qi-yin derives from the word ‘qi’, which, according to Traditional Chinese Medicine theory, is defined as the basic energy that maintains life activities. ‘Yin’ and ‘yang’ are considered to be the two opposing principles in nature, with ‘yin’ representing the feminine and negative and ‘yang’ representing the masculine and positive. A ‘qi-yin’ deficiency is associated with coronary heart disease, chronic pulmonary heart disease and viral myocarditis, in addition to heart and respiratory failure (15). According to Traditional Chinese Medicine theory, SM benefits qi, prevents exhaustion, nourishes yin and replenishes bodily fluids, while demonstrating protective effects against adverse drug reactions (16). SM is extracted from red ginseng and the ophiopogon root. Ginsenosides, the primary bioactive components of ginseng, are known to scavenge oxygen free radicals (17,18), block Ca^2+^ channels (17,19) and reduce the ischemia/reperfusion (I/R) injury associated with cardiovascular and cerebrovascular diseases (20–24). Previous studies have indicated that ginsenoside Rg1 protects cardiomyocytes under hypoxic conditions by reducing intracellular Ca^2+^ overload (24–26). Treatment with the total saponins of *Panax ginseng* (TSPG), i.e. the total quantity of ginsenosides extracted from ginseng, can increase cardiac SR Ca^2+^ levels, reduce mitochondrial Ca^2+^ levels and increase mitochondrial calcium pump activity (22,23). Furthermore, SM is able to mitigate apoptosis and Ca^2+^ influx in neurocytes following hypoxia-reoxygenation (21). It remains unclear, however, whether the myocardial protective effect of SM injection during I/R functions by maintaining the [Ca^2+^]_i_ via the regulation of PLB phosphorylation. An aim of the present study was to elucidate the mechanism underlying PLB involvement in each of these processes.

This study aimed to determine whether the myocardial protection from SM injection during I/R is associated with the maintenance of the [Ca^2+^]_i_ through the relief of PLB inhibition. In addition, a further aim was to determine whether the pharmacodynamic activity of SM injection is superior to that of the pure ginseng saponins compounds.

## Materials and methods

### 

#### Chemicals and reagents

SM was purchased from Chia Tai Qingchunbao Pharmaceutical Co., Ltd. (Hangzhou, China). Ginsenoside Rg1 (purity, >99%) was purchased from the Chinese National Institute for the Control of Pharmaceutical and Biological Products (Beijing, China). TSPG (purity, >80%) was purchased from Shanghai Winherb Medical Technology Co., Ltd. (Shanghai, China). Dulbecco's modified Eagle's medium (DMEM), fetal bovine serum (FBS) and TRIzol® reagent were obtained from Invitrogen Life Technologies (Carlsbad, CA, USA). Rabbit anti-PLB antibodies (#05-205) and ECL-Plus chemiluminescent substrate were purchased from EMD Millipore (Billerica, MA, USA). Mouse anti-phosphorylated PLB (p-PLB; phosphorylated at Thr 17 and Ser 16; #8496) and rabbit anti-SERCA antibodies (#9580) were purchased from Cell Signaling Technology, Inc. (Danvers, MA, USA). Mouse anti-β-actin (#CW0096) and horseradish peroxidase-labeled IgG secondary antibody were obtained from Beijing ComWin BioTech Co., Ltd. (Beijing, China). The PrimeScript™ RT reagent kit and SYBR® *Premix Ex Taq*™ II were from Takara Bio, Inc. (Otsu, Japan). Primers for the reverse transcription-quantitative polymerase chain reaction (RT-qPCR) were purchased from Sangon Biotech Co., Ltd. (Shanghai, China). The Annexin-V/propidium iodide (PI) Assay kit was purchased from BD Biosciences (Franklin Lakes, NJ, USA). Fluo-4-AM was purchased from Life Technologies.

#### Animals

The study was designed and all protocols involving animals were conducted according to the Guide for the Care and Use of Laboratory Animals published by the U.S. National Institutes of Health. In addition, the animal protocol utilized in this study was approved by the Ethics Committee of the Medical College of Zhejiang University (Hangzhou, China).

#### Primary cardiomyocyte culture

Cardiomyocytes were isolated from the hearts of neonatal Sprague Dawley rats that were ≤3 days old (SLAC Laboratory Animal Co., Ltd., Shanghai, China), and the isolated cells were cultured as described previously with minor modifications (27). To obtain the cardiomyocytes, heart tissue was digested with 0.1% collagenase type II (Invitrogen Life Technologies) and 0.125% pancreatin (Sigma-Aldrich, St. Louis, MO, USA) for 8 min at 37°C. Following centrifugation, the supernatant was discarded and the cell pellets were resuspended in culture media containing 10% FBS. These steps were repeated until the hearts were completely digested. Cells were pre-plated for 90 min to allow ﬁbroblasts to attach and to yield a purer cardiomyocyte population. The cardiomyocytes were maintained in DMEM containing 10% FBS, 0.1 mM 5-bromo-2′-deoxyuridine (Invitrogen Life Technologies) and 100 IU/ml 0.3% penicillin-streptomycin to inhibit the growth of other cell types. Following 3 days in culture, the cardiomyocytes were subjected to subsequent experimentation.

The cardiomyocytes were divided into the following eight groups: Normoxia (N), TSPG-treated normoxia (N + TSPG), Rg1-treated normoxia (N + Rg1), SM-treated normoxia (N + SM), I/R, TSPG-treated I/R (I/R + TSPG), Rg1-treated I/R (I/R + Rg1) and SM-treated I/R (I/R + SM).

#### Hypoxia/reoxygenation treatment protocol

Cardiomyocytes were pretreated with TSPG (1.5 mg/l), Rg1 (0.325 mg/l) or SM (5 ml/l) for 24 h. Cultured cardiomyocytes were subsequently washed with Hank's balanced salt solution (HBSS), containing 5 mM HEPES, 137 mM NaCl, 4 mM KCl, 1 mM MgCl_2_ and 1.5 mM CaCl_2_ (pH 7.2), and then incubated in glucose-free DMEM. Hypoxia was used to mimic the *in vivo* condition of myocardial ischemia. To induce hypoxia, the cells were placed in an incubator at 37°C, and then N_2_ (95%) and 5% CO_2_ were ﬂushed into the incubator to lower the oxygen concentration to 1%. Following 4 h of exposure to the hypoxic conditions, the cells were subjected to reoxygenation by exchanging the media with DMEM supplemented with 4.5 g/l glucose. Cells were then incubated under normoxic conditions for 1 h.

#### RNA analysis

Total RNA from the cardiomyocytes was isolated using TRIzol reagent according to the manufacturer's instructions. Total RNA concentration was determined photometrically using a wavelength of 260 nm. RNA samples were stored at −80°C. For the RT-qPCR, total RNA was transcribed using the PrimeScript RT reagent kit. The PCR reaction conditions were as follows: 95°C for 30 sec, then 40 cycles of 5 sec at 95°C and 34 sec at 60°C. Individual samples of 100 ng cDNA were amplified using SYBR *Premix Ex Taq* II, utilizing gene-specific primers in an ABI PRISM® 7500 Fast Sequence Detection System (Applied Biosystems, Foster City, CA, USA). Standard curves were performed in duplicate with serially diluted cDNA synthesized from neonatal rat heart tissue (1.5–50 ng) to determine the PCR efficiency, which yielded similar results in all groups. SERCA and PLB mRNA expression levels were evaluated as a ratio of SERCA/PLB. Quantification was performed using the standard curve and 2^−ΔΔCt^ methods.

The primers used for PCR analysis were as follows: SERCA2a forward, 5′-AAG CAG TTC ATC CGC TAC CT-3′ and reverse, 5′-AGA CCA TCC GTC ACC AGA TT-3′; PLB forward, 5′-TAC CTT ACT CGC TCG GCT ATC-3′ and reverse, 5′-TAC CTT ACT CGC TCG GCT ATC-3′; and β-actin forward, 5′-GGA GAT TAC TGC CCT GGC TCC TA-3′ and reverse, 5′-GAC TCA TCG TAC TCC TGC TTG CTG-3′.

#### Western blot analysis

Isolated cardiomyoctes were lysed in radioimmunoprecipitation assay buffer (50 mM Tris-HCl pH 7.4, 150 mM NaCl, 1% NP-40, 10.5% sodium deoxycholate, 5% sodium dodecyl sulfate and 1 mM phenylmethyl sulfonyl-ﬂuoride). The total protein concentration was quantiﬁed using a bicinchoninic protein quantity assay kit (Thermo Fisher Scientific, Inc., Waltham, MA, USA) and equal amounts of protein were loaded onto 12% sodium dodecyl sulfate-polyacrylamide gels. Following separation by electrophoresis, the proteins were electrotransferred onto polyvinylidene difluoride membranes. Non-specific binding sites were blocked by incubating with 5% skimmed milk in Tris-buffered saline containing 0.05% Tween-20 at room temperature for 1 h. The membranes were probed using rabbit anti-PLB (1:1,000), mouse anti-p-PLB (1:1,000), rabbit anti-SERCA2a (1:1,000) and mouse anti-β-actin (1:1,000) primary antibodies overnight at 4°C. The membranes were subsequently probed with horseradish peroxidase-labeled anti-rabbit IgG secondary antibody (1:10,000) at room temperature for 1 h. The immunoreactive bands were visualized using ECL-Plus reagent. Signal intensities of each band were analyzed using Quantity One® software, version 4.6.2 (Bio-Rad Laboratories, Inc., Berkeley, CA, USA), and the relative protein levels were calculated by comparing with the β-actin loading control.

#### Annexin V/PI assay

Briefly, cardiomyocytes were collected, washed with Ca^2+^-free phosphate-buffered saline, resuspended in binding buffer and incubated with 5 µl Annexin V and PI at room temperature in the dark for 15 min. The cardiomyocytes were then analyzed using a ﬂow cytometer (FC500MCL; Beckman Coulter, Brea, CA, USA).

#### Measurement of [Ca^2+^]_i_

After 1 h of reoxygenation, [Ca^2+^]_i_ measurements were conducted using the Ca^2+^-sensitive ﬂuorescent probe ﬂuo-4 AM. Cardiomyocytes were incubated in six-well plates with 5 µM ﬂuo-4 AM for 30 min at 37°C. The cells were washed with HBSS three times. Fluorescence levels were measured using ﬂow cytometry, with excitation at 484 nm and emission at 516 nm.

#### Statistical analysis

Statistical analyses were performed using SPSS software, version 19.0 (IBM SPSS, Armonk, NY, USA). Statistical significance was detected using one-way analysis of variance and Student's *t*-tests. P<0.05 was considered to indicate a statistically signiﬁcant difference.

## Results

### 

#### Effects of TSPG, Rg1 and SM on SERCA and PLB mRNA expression levels

**Figure 1.**
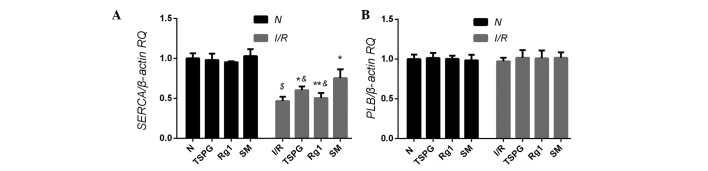
Effects of TSPG, Rg1 and SM on SERCA and PLB mRNA expression following I/R in cardiomyocytes. (A) SERCA and (B) PLB mRNA expression levels. Data are expressed as the mean ± standard deviation (n=9). ^$^P<0.01 vs. N; ^&^P<0.01 vs. I/R + SM; *P<0.01 vs. I/R; **P<0.01 vs. I/R+TSPG. SERCA, sarco/endoplasmic reticulum Ca^2+^ ATPase; PLB, phospholamban; N, normoxia; RQ, relative quantification; I/R, ischemia/reperfusion; TSPG, total saponins of *Panax ginseng*; Rg1, ginsenoside Rg1; SM, Shenmai injection.

In order to determine whether TSPG, Rg1 or SM treatment was able to affect SERCA and PLB mRNA expression, rat myocardial cells were subjected to I/R, and the resulting mRNA levels were compared with those of the normoxic cells. No significant difference in the PLB mRNA levels was detected between the I/R group and the untreated controls (P>0.05); however, the SERCA mRNA levels were found to be significantly decreased in the I/R group (P<0.01). Treatment of the I/R cells with TSPG (I/R + TPSG) or SM (I/R + SM) resulted in a signiﬁcant upregulation of SERCA mRNA levels, with SM treatment showing the most marked effect (P<0.01). Notably, this difference in mRNA expression was not evident in the I/R + Rg1 group (P>0.05) (Fig. 1).

#### Effects of TSPG, Rg1 and SM on SERCA, PLB and p-PLB protein levels

In addition to the mRNA analysis, this study aimed to determine whether TSPG, Rg1 or SM treatment was able to affect SERCA, PLB and/or p-PLB protein expression. Following I/R treatment, the PLB protein levels in the I/R treatment group were compared with those in the N group, and no statistically significant difference was detected (P>0.05). Conversely, the protein levels of p-PLB and the p-PLB/PLB ratio were significantly reduced in the I/R group compared with those in the N group (both P<0.01); however, the I/R + SM group exhibited a significant increase in p-PLB protein levels (P<0.01), in addition to an increase in the p-PLB/PLB expression ratio, compared with the I/R group (P<0.01). Notably, the two other drug administration groups (TSPG and Rg1) showed no signiﬁcant alterations in p-PLB protein expression (P>0.05) or p-PLB/PLB ratio (P>0.05).

**Figure 2.**
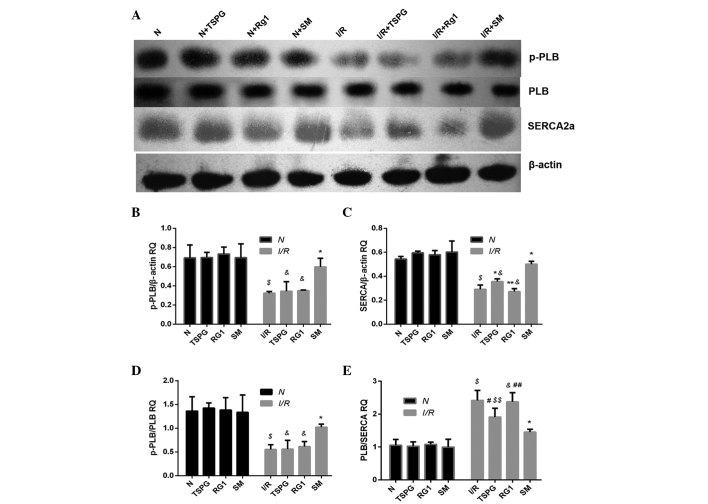
Effects of TSPG, Rg1 and SM on SERCA, p-PLB, p-PLB/PLB and PLB/SERCA levels following I/R in cardiomyocytes. (A) Western blot analysis of SERCA, p-PLB, PLB and β-actin levels. (B-E) Quantification of (B) p-PLB levels, (C) SERCA levels, (D) p-PLB/PLB ratio and (E) PLB/SERCA ratio. Data are expressed as the mean ± standard deviation (n=4). ^$^P<0.01 vs. N; ^&^P<0.01 and ^$$^P<0.05 vs. I/R + SM; *P<0.01 and ^#^P<0.05 vs. I/R; **P<0.01 and ^##^P<0.05 vs. I/R + TSPG. N, normoxia; TSPG, total saponins of *Panax ginseng*; Rg1, ginsenoside Rg1; SM, Shenmai injection; I/R, ischemia/reperfusion; PLB, phospholamban; p-PLB, phosphorylated-PLB; SERCA, sarco/endoplasmic reticulum Ca^2+^ ATPase; RQ, relative quantification.

The comparison of SERCA protein levels between the N and I/R treatment groups revealed a signiﬁcant reduction following I/R treatment (P<0.01). Conversely, the SERCA protein expression levels were signiﬁcantly elevated in the I/R + SM and I/R + TSPG groups compared with the levels in the I/R group (P<0.01), whereas the SERCA protein expression in the I/R and I/R + Rg1 groups showed no significant difference (P>0.05). The PLB/SERCA ratio was signiﬁcantly higher in the I/R group compared with that in the N group (P<0.01), while this ratio was signiﬁcantly decreased in the I/R + SM (P<0.01) and I/R + TSPG (P<0.05) groups. The I/R + Rg1 group exhibited a modest but insignificant reduction in the PLB/SERCA ratio (P>0.05). Notably, the I/R + SM group showed the most marked increase in SERCA protein levels and the most marked reduction in the PLB/SERCA ratio compared with the I/R + TSPG group (P<0.05/0.01) (Fig. 2).

#### Effects of TSPG, Rg1 and SM on cardiomyocyte apoptosis following I/R injury

**Figure 3.**
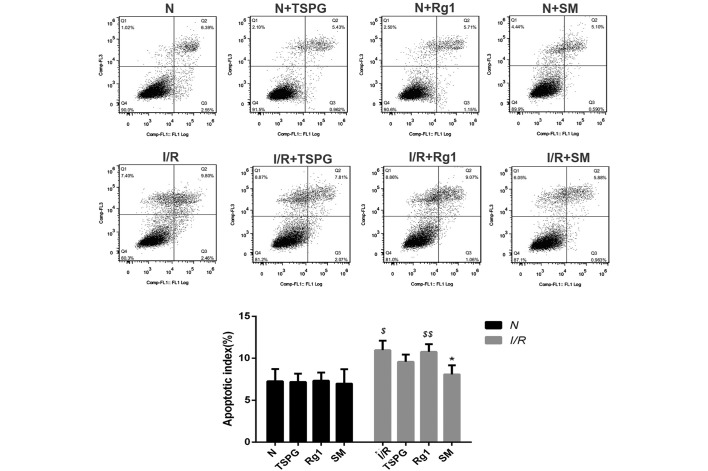
Effects of TSPG, Rg1 and SM on I/R-induced cardiomyocyte apoptosis. Cardiomyocytes in regions Q3 and Q2 represent early- and late-apoptotic cardiomyocytes, respectively. Data are expressed as the mean ± standard deviation (n=3). ^$^P<0.01 vs. N; ^$$^P<0.05 vs. I/R + SM; *P<0.01 vs. I/R. N, normoxia; TSPG, total saponins of *Panax ginseng*; Rg1, ginsenoside Rg1; SM, Shenmai injection; I/R, ischemia/reperfusion.

In order to examine the effects of TSPG, Rg1 and SM treatment on I/R-induced apoptosis, the apoptosis rates were determined using ﬂow cytometry. As shown in Fig. 3, I/R treatment markedly increased the apoptosis rates (P<0.01); however, this effect was attenuated with SM treatment (P<0.05). In addition, the I/R + Rg1 group showed a modest, insignificant reduction in apoptosis (P>0.05), and the I/R + TSPG group exhibited a greater but also insignificant reduction in apoptosis (P>0.05) (Fig. 3).

#### Effects of TSPG, Rg1 and SM on [Ca^2+^]_i_ in cardiomyocytes following I/R injury

**Figure 4.**
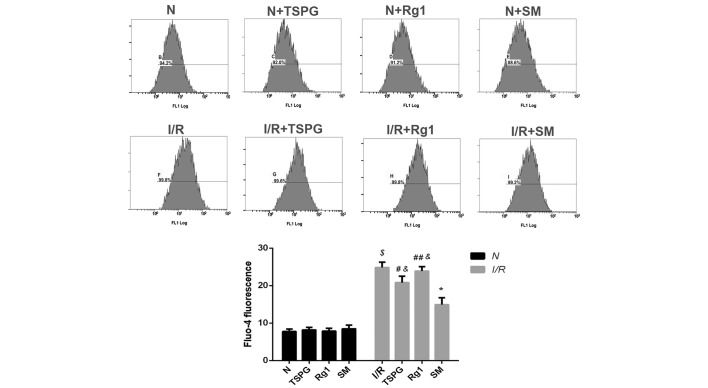
Effects of TSPG, Rg1 and SM on the intracellular Ca^2+^ concentration in cardiomyocytes following I/R. Data are expressed as the mean ± standard deviation (n=3). ^$^P<0.01 vs. N; ^&^P<0.01 vs. I/R + SM; *P<0.01 and ^#^P<0.05 vs. I/R; **P<0.01 and ^##^P<0.05 vs. I/R + TSPG. N, normoxia; TSPG, total saponins of *Panax ginseng*; Rg1, ginsenoside Rg1; SM, Shenmai injection; I/R, ischemia/reperfusion.

As shown in Fig. 4, I/R markedly increased the [Ca^2+^]_i_ (P<0.01); however, this increase was suppressed by the SM (P<0.01) and TSPG (P<0.05) treatments. The I/R + Rg1 group exhibited a modest but insignificant decrease in [Ca^2+^]_i_ compared with the I/R group (P>0.05). The I/R + SM group showed the greatest decrease in [Ca^2+^]_i_ following I/R treatment (P<0.01) (Fig. 4).

## Discussion

In the present study, the mechanism underlying the myocardial protective effect of SM following I/R was investigated. It was demonstrated that exposure to I/R resulted in significant alterations in a variety of functional indices for cardiac function, including a disruption in Ca^2+^ transport within heart myocardial cells. In accordance with a previous study, the apoptosis rate and [Ca^2+^]_i_ of cardiomyocytes increased significantly following I/R, indicating that I/R injury successfully occurred (28).

In order to further clarify the effects of SM on myocardial cells, three different drug treatments were employed to determine their comparative efficacies in abating I/R-related complications. Ginsenoside Rg1 is a major bioactive component of TSPG, while TSPG is the primary bioactive component of SM. It has been established that 1 ml SM contains 65±6.68 µg Rg1 and 293.38±40.54 µg TSPG (29). In accordance with similar studies concerning drug treatment of cardiomyocytes (30,31), 5 ml/l SM was administered in the present treatment regimen, which is equivalent to ~1.5 mg/l TSPG and ~0.325 mg/l Rg1. PLB, the primary regulator of SERCA, is a small transmembrane SR protein present in the ventricles of the heart and, to a lesser extent, in the atria (32). Signiﬁcant alterations in the stoichiometry between SERCA and PLB have been associated with chronic heart failure. PLB therefore functions as a physiological brake on excitation-contraction coupling, therein modulating cardiac function. The present results indicated that the reduced p-PLB expression following I/R was restored to normal levels following SM treatment, but not following treatment with TSPG or Rg1. This observation supports the conclusion that the ability of SM to counteract the negative effects of I/R treatment is partially attributable to increased PLB phosphorylation, but not to TSPG or Rg1 when administered at similar concentrations.

The SR Ca^2+^-ATPase is responsible for restoring the SR Ca^2+^ load per excitation-contraction cycle. A reduction in SERCA content is associated with reduced SR Ca^2+^ loading and elevated [Ca^2+^]_i_ (33,34). The restoration of SERCA levels is potentially a critical factor in normalizing Ca^2+^ uptake, as observed in line-scan and frequency-dependent experiments (35). It is therefore plausible that the observed reduction in SERCA expression may partially explain the observed [Ca^2+^]_i_ overload in cardiomyocytes following I/R treatment. The present study has demonstrated that SM and TSPG are able to increase SERCA expression and that this improves intracellular Ca^2+^ cycling. Notably, the numerous changes observed following I/R treatment were not reversed by Rg1 treatment, which is inconsistent with the results of previous studies (25,26). This discrepancy may be attributable to the different doses of Rg1 used, as our study utilized a dose far below proven efficacy. To the best of our knowledge, the present study is the first to conduct a comparison between the effects of SM and its purified constituent compounds on the expression of p-PLB. Notably, the ratio of p-PLB/PLB levels was significantly reduced in the I/R group relative to that in the control N group (P<0.01); therefore, despite PLB expression remaining unaltered following I/R treatment, p-PLB expression decreased significantly.

In conclusion, the myocardial protective effects of SM following I/R treatment may be partially attributable to the effects of SM on intracellular Ca^2+^ homeostasis, specifically the relief of PLB inhibition. Notably, the present results indicate that the pharmacodynamic activity of TPSG is significantly superior to that of Rg1; however, the effects of SM are significantly superior to those of TSPG and Rg1.
